# Atrioventricular Ring Tachycardias: Atypical Fast-Slow Atrioventricular Nodal Reentrant Tachycardia and Atrial Tachycardia Share a Common Arrhythmogenic Substrate—A Unifying Proposal

**DOI:** 10.31083/j.rcm2311369

**Published:** 2022-10-28

**Authors:** Yoshiaki Kaneko, Shuntaro Tamura, Takashi Kobari, Hiroshi Hasegawa, Tadashi Nakajima, Hideki Ishii

**Affiliations:** ^1^Department of Cardiovascular Medicine, Gunma University Graduate School of Medicine, Maebashi, 371-8511 Gunma, Japan

**Keywords:** atrioventricular nodal reentrant tachycardia, slow pathway, tricuspid annulus, atrial tachycardia, atrioventricular ring, adenosine sensitivity

## Abstract

Our understanding of the variants of slow pathway (SP) and associated atypical atrioventricular (AV) nodal reentrant tachycardia (NRT) is still growing. We have identified variants extending outside Koch’s triangle along the tricuspid annulus, including superior, superoanterior and inferolateral right atrial SP and associated atypical, fast-slow AVNRT. We review the history of each variant, their electrophysiological characteristics and related atypical AVNRT, and their treatment by catheter ablation. We focused our efforts on organizing the published information, as well as some unpublished, reliable data, and show the pitfalls of electrophysiological observations, along with keys to the diagnosis of atypical AVNRT. The superior-type of fast-slow AVNRT mimics adenosine-sensitive atrial tachycardia originating near the AV node and can be successfully treated by ablation of a superior SP form the right side of the perihisian region or from the non-coronary sinus of Valsalva. Fast-slow AVNRT using a superoanterior or inferolateral right atrial SP also mimics atrial tachycardia originating from the tricuspid annulus. We summarize the similarities among these variants of SP, and the origin of the atrial tachycardias, including their anatomical distributions and electrophysiological and pharmacological characteristics. Moreover, based on recent basic research reporting the presence of node-like AV ring tissue encircling the annuli in adult hearts, we propose the term “AV ring tachycardia” to designate the tachycardias that share the AV ring tissue as a common arrhythmogenic substrate. This review should help the readers recognize rare types of SP variants and associated AVNRT, and diagnose and cure these complex tachycardias. We hope, with this proposal of a unified tachycardia designation, to open a new chapter in clinical electrophysiology.

## 1. Introduction

The concept of atrioventricular (AV) nodal reentrant tachycardia (NRT) is still evolving, as variations in slow pathway (SP) are being clarified. Dual AV nodal conduction was first conceptualized on the basis of electrophysiological experiments in canine hearts, performed by Gordon Moe and his collaborators [1]. Shortly thereafter, clinical electrophysiologic studies with recordings of the His bundle activation, along with programed stimulation of the heart, were developed. Based on the observation of discontinuous AV conduction during atrial extrastimulation, the existence of dual AV nodal conduction was confirmed in humans and was proposed as the electrophysiological substrate of slow-fast AV nodal reentry [2], while fast-slow AVNRT was also reported nearly simultaneously [3]. However, at that time, the precise location of the SP was uncertain and inaccurately placed inside the AV node, on top of Koch’s triangle. The first sign that the atrial end of the SP was away from the fast pathway (FP) was recognized during a clinical electrophysiologic study, when an electrode catheter was used to record from inside the coronary sinus (CS) [4]. During that study, the site of earliest atrial activation during retrograde conduction over the SP was recorded at the proximal CS. This observation revolutionized the concept of dual AV nodal pathways and opened the way to the cure of AVNRT by the surgical dissection of the SP [5], followed by its selective catheter ablation, keeping antegrade conduction intact over the FP [6, 7]. The successful elimination of the SP with these techniques confirmed that it was located in the posterior septum. Thereafter, a right inferior extension of the SP was anatomically confirmed [8]. Subsequent discoveries of variants, including a left [9, 10] and an inferolateral left atrial SP [11] were also based on clinical investigations, without anatomic confirmations.

The concepts of SP and AVNRT have, therefore, consistently been based on clinical electrophysiological observations, followed by the anatomical confirmation of the substrate, representing an unprecedented historic evolution. By applying electrophysiological techniques, we have recently identified several variants of SP extending into the tricuspid annulus and associated AVNRT.

We review and discuss here these SP and associated AVNRT, and propose new tachycardia entities, including AVNRT, based on their presumed arrhythmogenic substrate.

## 2. Variants of SP Extending to the Tricuspid Annulus and Their Associated Atypical Reentrant Tachycardias

### 2.1 Superior or Superoanterior SP

#### 2.1.1 Historical Overview

The superior SP is a variant extending superiorly outside Koch’s triangle, which can be confirmed by electrophysiological observations of (1) the earliest site of retrograde atrial activation above the His bundle region, or (2) its successful selective ablation near the right side of the AV node or the non-coronary sinus of Valsalva, or (3) both [1]. We have described a distinct “superior-type” entity of fast-slow AVNRT using a superior SP as its retrograde limb [12]. However, before our report, several investigators had also hypothesized the existence of a substrate localized on top of the Koch’s triangle, responsible for atypical AVNRT. DiMarco *et al*. [13] reported a case of atypical AVNRT, with the earliest atrial site of activation located in the His bundle region and Wenckebach type AV block, and presumed that the reentry circuit was within the upper portion of the AV node, based on the responses of the tachycardia to atrial stimulation. This might have been the first published case of superior-type fast-slow AVNRT. Using intraoperative ice mapping Keim *et al*. [14] identified the first case of slow-fast AVNRT, where the SP extended superiorly into the interatrial septum. No case has since been reported of slow-fast AVNRT using a superior SP as the anterograde limb. In 3 cases of atypical fast-slow AVNRT, Nawata *et al*. [15] hypothesized the existence of a SP extending above the His bundle as a retrograde limb of the circuit. However, they did not proceed with ablation to confirm the presence of the SP. Cases reported by Otomo *et al*. [16] of atypical fast-slow AVNRT with a site of earliest atrial activation in the His bundle region, eliminated by ablation at the midseptal level, were probably similar to our “superior-type” of AVNRT. Lockwood *et al*. [17] also described, in the same period, the presence of an anterosuperior SP and associated atypical slow-slow or fast-slow AVNRT using this SP variant as the retrograde limb. No further case of superior SP has been reported, until our case where a superior SP was successfully ablated in the non-coronary cusp of the aortic valve [18].

**Fig. 1.**
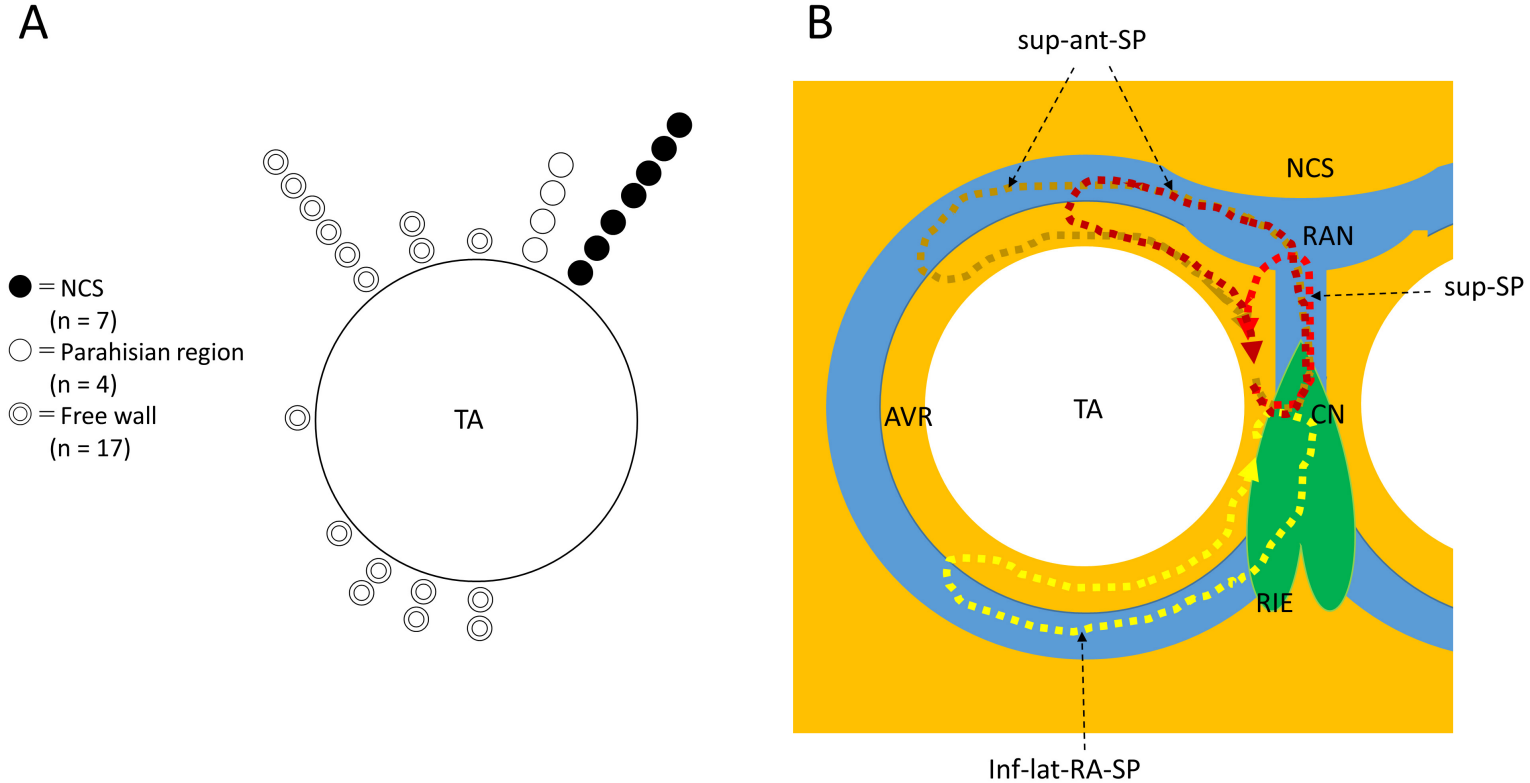
**Distribution of the site of earliest atrial activation during fast-slow AVNRT using a superior, superoanterior or inferolateral right atrial SP (A) and schematic illustration of the reentry circuits of an atypical fast-slow AVNRT using variants of SP, the AV ring (AVR) and the retroaortic node (RAN) (B)**. NCS, non-coronary sinus of Valsalva, TA, tricuspid annulus. (B) The AVR encircling the TA is continuous with the right inferior extension (RIE) of the AV node and with the RAN, just behind to NCS. The inferolateral right atrial slow pathway (inf-lat-RA-SP) is formed by the continuous RIE and AVR, and is used as the retrograde limb of the reentrant inferolateral-type of fast-slow AVNRT. The superior slow pathway (sup-SP), formed by nodal-like tissue connecting the compact node (CN) and RAN, is used as the retrograde limb for the reentrant superior-type of fast-slow AVNRT. The superoanterior slow pathway (sup-ant-SP) is formed by the AVR in continuity with the RAN and the components of sup-SP, and is used as a retrograde limb of the reentry circuit of the superoanterior-type of fast-slow AVNRT.

We have, thus far, studied over 20 cases of superior-type fast-slow AVNRT (Fig. 1A). In this series, we have observed sites of earliest atrial activation and successful ablation in the superoanterior region of the right atrial free wall, along the tricuspid annulus, suggesting the presence of a “superoanterior” SP extending to the superoanterior right atrial wall [19, 20]. In some of these cases, retrograde conduction via the superoanterior SP was reproducible with ventricular stimulation [20] and occasionally revealed multiple atrial exits.

Several histological studies support the presence of a superior or superoanterior SP. Inoue *et al*. [21] identified node-like tissue originating from the compact AV node and extending superiorly, named “superior extension”. Kato *et al*. [22] found an aggregation of node-like cells around the annulus in all hearts they studied. The genesis of the superior SP will be discussed later.

#### 2.1.2 Electrophysiological Diagnosis of Associated Atypical AVNRT

The superior-type of fast-slow AVNRT, the main subtype of AVNRT using a superior SP, is characterized by a long RP and a site of earliest atrial activation above the His bundle region during tachycardia [12]. Therefore, the electrophysiological diagnosis of this AVNRT is made by excluding (a) an atrial tachycardia originating in the vicinity of the AV node, and (b) AV reentrant tachycardia using a slowly conducting accessory pathway running in the perihisian region [12]. A diagnosis of AV reentrant tachycardia can be easily excluded by the observation of ventriculoatrial (VA) dissociation during ventricular overdrive pacing of the tachycardia [23, 24] or no change in the atrial cycle during the transition of QRS complexes immediately after ventricular overdrive pacing of the tachycardia [12, 25, 26]. The former, although not specific, is often observed during ventricular overdrive pacing of the superior-type, fast-slow AVNRT, probably because the retrograde conductivity of the lower common pathway decreases as the tachycardia develops, inhibiting the retrograde penetration into the AV nodal reentry circuit [12]. The latter may never occur with AV reentrant tachycardia using a right septal accessory pathway, due to the repetitive or fully premature retrograde penetration of the accessory pathway in the QRS transition zone during ventricular pacing from the site ipsilateral to the accessory pathway [27]. This is in contrast with AV reentrant tachycardia using a left lateral accessory pathway, where a false negative response is occasionally observed [27]. Moreover, the atrial preexcitation phenomenon typically diagnostic of AV reentry [28] and evidenced by atrial resetting of the tachycardia by a single premature ventricular stimulus delivered during His bundle refractoriness, may be absent if the tachycardia uses a slowly conducting accessory pathway, because of the decremental conduction caused by the ventricular stimulus [29]. Therefore, instead of single premature ventricular extrastimuli delivered during the tachycardia, we use ventricular overdrive pacing to differentiate atypical AVNRT from AV reentrant tachycardia and orthodromic reentrant tachycardia using concealed nodo-ventricular or nodo-fascicular fibers [30].

In contrast, the discrimination of sup-F/S-AVNRT versus atrial tachycardia remains challenging. Although the observations of (a) a V-A-V response upon ventricular induction/entrainment [31], and (b) termination of the tachycardia by ventricular pacing without atrial capture [32] are most useful to exclude the diagnosis of atrial tachycardia. The likelihood of these observations is low because of retrograde block occurring in the lower common pathway [12]. In fact, a V-A-V response upon ventricular entrainment is rare [16, 33]. Instead, one is more likely to observe several electrophysiological phenomena characteristic of this type of AVNRT. Understanding these phenomena facilitates a rapid and accurate diagnosis.

*First*, in AV nodal reentry, a strong link is believed to be present between ventricular and subsequent atrial activation during differential atrial entrainment pacing from multiple sites in the atria [34]. However, when pacing a superior-type of fast-slow AVNRT, VA linking may be occasionally absent, which can be recognized by a shorter VA interval after entrainment pacing from the high right atrium (HRA) than from the proximal CS [35]. This may cause an erroneous diagnosis of atrial tachycardia and is explained by a pacing site-dependent shortening of the retrograde conduction time over the SP, immediately after entrainment pacing. Indeed when the wavefront reaches earlier and penetrates deeper into the atrial end of the SP during entrainment pacing from site A than from site B, depending on the physical relationship between the site of pacing and dual AV nodal pathways, the subsequent retrograde conduction time over the SP is shorter after pacing from A than from B, due to decremental conduction [35].

*Second*, a dual atrial response resulting from simultaneous retrograde conduction over the fast and superior SP, causing a V-A-A-V response, is often observed, especially upon ventricular induction of a superior-type of fast-slow AVNRT [36]. Therefore, based on characteristics of the V-A-A-V response, a tachycardia with an earliest site of atrial activation in the His bundle region is an atrial tachycardia. We believe that the inter-electrograms analysis of the V-A-A-V response partially allows the discrimination between fast-slow AVNRT and atrial tachycardia: when the interatrial interval of the V-A-A-V response minus the tachycardia cycle length is defined as ΔAA, a ΔAA >26 ms is diagnostic of fast-slow AVNRT (the V-A-A-V response being due to a dual atrial response), whereas a ΔAA <–80 ms is diagnostic of atrial tachycardia [30, 36].

**Fig. 2.**
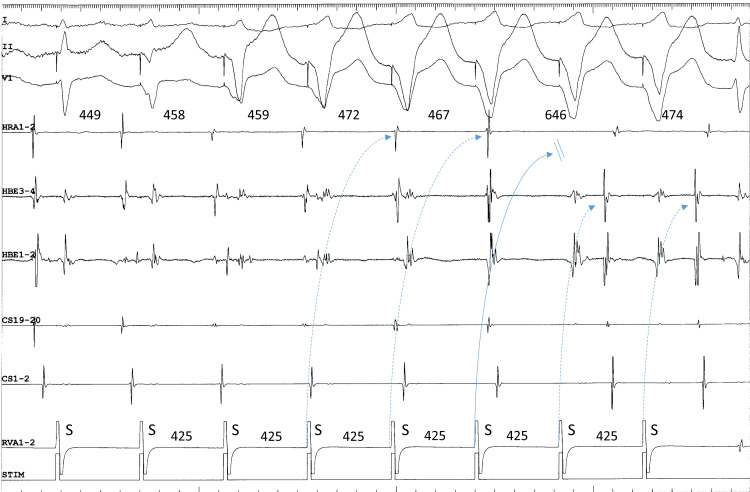
**Termination of fast-slow AVNRT using a superoanterior SP during ventricular overdrive pacing at an S-S cycle length of 425 ms from the right ventricular apex (RVA1-2)**. The site of earliest atrial activation during tachycardia is recorded in the HRA (HRA1-2). The atrial cycle length immediately after the 4th and 5th stimuli lengthens slightly without change in the atrial activation sequence, consistent with an orthodromic capture of the atria over the superoanterior SP, with a decremental delay in response to the 4th and 5th stimuli (dotted arrows). The 6th stimulus is blocked (straight arrow), evidenced by the absence of retrograde activation over the SP in its wake. In response to the 6th and 7th ventricular stimuli, the site of earliest retrograde atrial activation was observed in the distal electrogram of the His bundle region (HBE1-2) along with a short ventriculoatrial interval, consistent with retrograde conduction over a FP. The numbers above the HRA1-2 channel are the interatrial intervals. I, II and $V_{1}$, surface electrocardiogram leads; CS 19-20 to 1-2, proximal to distal CS.

*Third*, when the tachycardia is terminated by ventricular overdrive pacing, the atrial cycles immediately before the termination may lengthen transiently, due to repetitive retrograde conduction with a decremental delay over the superior SP, followed by orthodromic block inside the superior SP (Fig. 2). This is not simple ventricular entrainment nor termination without atrial capture, but a frequently observed, confirmatory, diagnostic phenomenon which excludes the diagnosis of atrial tachycardia.

**Fig. 3.**
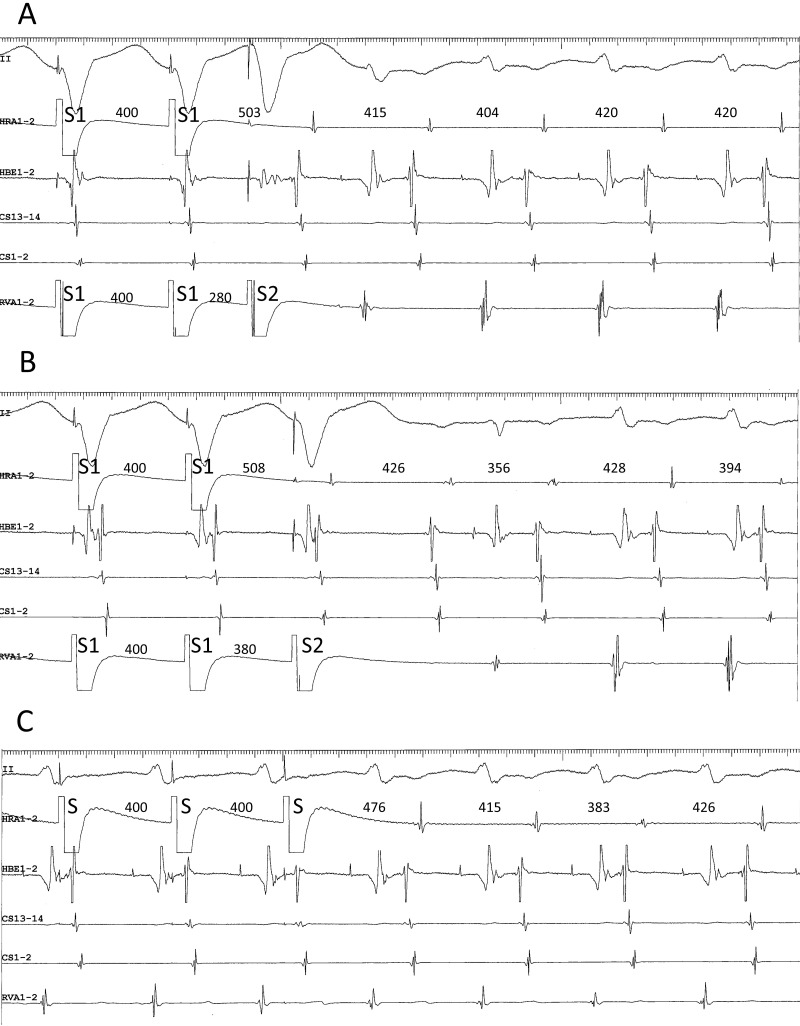
**(A) pseudo-V-A-V and (B) atypical V-A-A-V responses observed upon initiation of the tachycardia after ventricular extrastimuli (S2) at S1-S2 coupling intervals of 280 (A) and 380 ms (B), following trains of simultaneous atrial and ventricular pacing at an S1-S1 cycle length of 400 ms, in a patient presenting with a superior-type of fast-slow AVNRT**. Ventriculoatrial conduction was absent at baseline. In both (A) and (B), the interval between the last atrial S1 and the first atrial electrogram of the tachycardia is similar to the interval between the last atrial S and the first atrial electrogram of the tachycardia induced by regular atrial pacing at an S-S cycle length of 400 ms (C). Therefore, the tachycardia is presumed to be induced by the atrial pacing train and follow the ventricular S2, instead of being induced by the ventricular S2. This resulted in an apparent V-A-V, or pseudo-V-A-V (A), and atypical V-A-A-V responses due to anterograde block in the lower common pathway caused by its refractoriness in the wake of S2 (B). II, surface electrocardiogram lead II; HRA1-2, high right atrium; HBE1-2, His bundle electrogram; CS13-14 to 1-2, proximal to distal coronary sinus; RVA1-2, right ventricular apex.

*Fourth*, ventricular extrastimulation after simultaneous stimulation of atrium and ventricle is useful to induce the superior-type of fast-slow AVNRT by exposing or accelerating a fragile retrograde conduction over the lower common pathway; however, this may cause a complex sequence of AV or VA activation, possibly leading to a misinterpretation of the AV or VA relationship. For example, when the tachycardia follows the last ventricular stimulus (Fig. 3A), it appears to be induced by that stimulus, creating a “pseudo-V-A-V response” (Fig. 3A), when in fact it was induced by atrial stimulation (Fig. 3A). Furthermore, when anterograde conduction of the 1st cycle of the induced tachycardia encounters the refractoriness produced by the last ventricular stimulus in the lower common pathway, the VA relationship upon induction reveals a V-A-A-V response (Fig. 3B) [37].

*Fifth*, when the tachycardia is induced by atrial stimulation, AV block may occur only in the 1st cycle of the induced tachycardia due to anterograde block in the lower common pathway, thus representing an initial A-A-V activation sequence [19, 38]; however, this activation sequence is also observed upon the induction of atrial tachycardia and, therefore, is not specific.

Electro-anatomical activation mapping of tachycardia in the right atrium and, if possible, in the non-coronary sinus of Valsalva is mandatory to identify the precise location of the site of earliest atrial activation, which is presumed to be the atrial end of the superior SP. A particularly meticulous mapping should be performed along the tricuspid annulus to identify the presumed atrial end of a superoanterior SP. Albeit rare, the right-sided interatrial septum away from the tricuspid annulus may be the site of earliest atrial activation during tachycardia.

Since we first reported the superior-type, fast-slow AVNRT [12], several cases of this tachyarrhythmia have recently been reported by other Japanese [39, 40, 41] as well Chinese [42] investigators. In contrast, few reports of this AVNRT have hailed from western countries [43], probably because it is underdiagnosed as atrial tachycardia originating from the vicinity of the AV node, in absence of universal diagnostic criterion to discriminate it from atrial tachycardia. Further studies are needed to find new diagnostic criteria and resolve these issues.

Atypical, slow-slow AVNRT is another subtype of AVNRT using a superior SP in a retrograde direction [44, 45]. The electrophysiological manifestations of this subtype is (a) a long AH interval, (b) an earliest site of atrial activation above the His bundle, and (c) its successful elimination by ablation of a typical SP, mimicking the manifestations of typical slow-fast AVNRT [44, 45]. Its prevalence, though difficult to determine precisely, is probably <1% of all slow-slow AVNRT [45]. An apparent electrophysiological prerequisite for its development is fragile anterograde conduction properties of the FP, preventing a participation of the latter as the anterograde limb of AVN reentry, in contrast to its participation in the superior-type, fast-slow AVNRT. On the other hand, the observation during an electrophysiologic study of a slow-fast AVNRT using a superior SP as the anterograde limb has never been reported, besides a single case successfully treated during intraoperative cryomapping [14]. Simple differential atrial entrainment pacing may be a valid means of screening this atypical slow-fast AVNRT [46]. When ablation of a typical SP fails to eliminate an apparent slow-fast AVNRT, cryomapping should be used, in search of a variant of SP as the anterograde limb, including a superior SP.

#### 2.1.3 Catheter Ablation

**Fig. 4.**
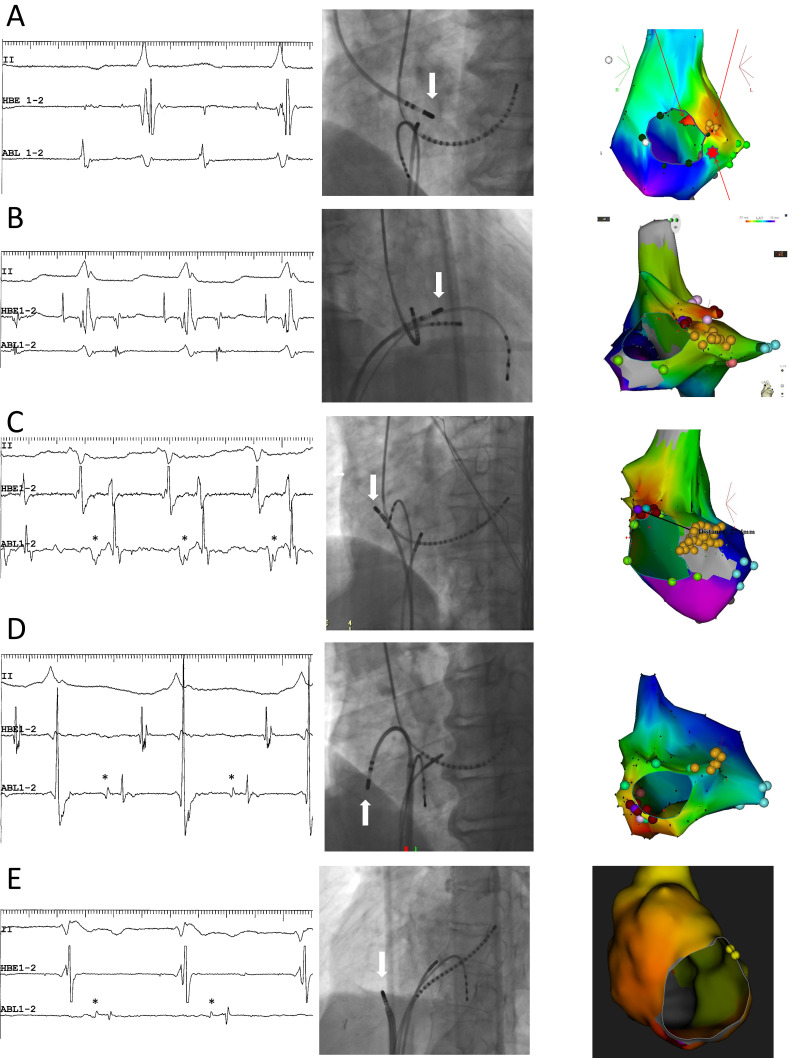
**Representative examples**. (A,B) Superior-type of fast-slow AVNRT successfully ablated in the non-coronary sinus of Valsalva (A) and the right perihisian region (B). (C) Superoanterior type fast-slow AVNRT. (D) Inferolateral type fast-slow AVNRT: E: ATP-sensitive atrial tachycardia originating from the inferolateral right atrial wall, along the tricuspid annulus. Left panels (A) to (E): intracardiac electrograms during tachycardia at the site of successful ablation. The asterisks in C, D and E mark the prepotentials preceding the local atrial electrograms. II, surface electrocardiogram lead II; HBE1-2, His bundle electrogram; ABL1-2, distal pole of ablation catheter. Middle panels: right (B) and left (A,C,D,E) anterior oblique fluoroscopic views of the catheter positions at the site of successful ablation. The white arrow points to the tip of the ablation catheter. Right panels: left anterior oblique views of three-dimensional activation maps of the right atrium during tachycardia, using ${\mathrm{CARTO}}^{\text{TM}}$ (A to D) and ${\mathrm{Rhythmia}}^{\text{TM}}$ (E) systems. The yellow tags indicate the sites of recording of the His bundle electrogram. The middle panel of Fig. 4B is reproduced from Kaneko *et al*. [19] (Copyright © 2016, the American Heart Association), and the left, middle and right panels of Fig. 4D are reproduced from Kaneko *et al*. [47] (Copyright © 2019, the Japanese Circulation Society), with permission from the publishers.

The superior-type fast-slow AVNRT is curable by ablation of the superior SP. In general, the site of earliest retrograde atrial activation over the superior SP during tachycardia or during ventricular stimulation must be targeted to ablate its atrial insertion. However, if the site of earliest atrial activation cannot be located either in the right perihisian region or in the non-coronary sinus of Valsalva, due to a close earliness of atrial activation, the latter can be targeted as the first site of ablation attempt to avoid injury to the AV node. The delivery of radiofrequency energy to the non-coronary sinus of Valsalva should be limited to the site of recording of an atrial electrogram of the highest amplitude as possible (Fig. 4A, Ref. [19, 47]), since, in these cases, the atrial end of the superior SP is probably inserted into the interatrial septum. Successful applications of radiofrequency energy terminate the tachycardia by retrograde block in the superior SP, usually without development of an accelerated junctional rhythm. If several applications remain unsuccessful at that site, the target of ablation may be moved to the right perihisian region. To keep the site of ablation away from the compact node and minimize the risk of AV nodal injury, the target should be limited to immediately behind the membranous septum, above the level of His bundle activation, where the near-field atrial and far-field ventricular electrograms are present and no His bundle electrogram is visible (Fig. 4B).

Ablation of the superoanterior SP should begin at the site of earliest atrial activation, corresponding to its atrial end (Fig. 4C), as its precise trajectory, especially between its atrial end and the compact AV node, remains unidentified. Each radiofrequency application often causes a shift in the site of earliest atrial activation; therefore, multiple applications may be needed to treat each new site of earliest activation. This phenomenon suggests that the SP widens at its atrial end. More interestingly, low-frequency potentials preceding the local atrial activation are often detectable near the site of earliest atrial activation (Fig. 4C), probably reflecting retrograde activation of the superoanterior SP. These electrophysiological observations contrast with the superior SP, the retrograde activation of which is not always detectable. Intracardiac echocardiography may be useful to navigate the tip of the ablation catheter in the non-coronary sinus of Valsalva [48, 49, 47], the perihisian region [50] or along the tricuspid annulus [51, 52].

### 2.2 Right Inferolateral Atrial SP 

#### 2.2.1 Historical Overview

Since Sung *et al*. [4] recorded at the CS ostium the site of earliest atrial activation during retrograde conduction across a typical SP, its atrial end has been believed to be located near that ostium. Moreover, even accounting for interindividual variations in the length of the typical SP functioning as the actual pathway [53, 54], or in the location of the compact node itself [55], the typical SP is generally believed to be located within Koch’s triangle [56, 57]. However, this is not based on a precise identification of the atrial end of the typical SP at the site of earliest atrial activation during retrograde conduction.

When performing activation mapping of the right atrium during fast-slow AVNRT, we observed seven cases where the site of earliest atrial activation was in the inferior or inferolateral right atrial free wall, along the tricuspid annulus (Figs. 1,4D) [58]. Convinced of our diagnosis of fast-slow AVNRT, we named “inferolateral right atrial SP” this variant of SP with a breakthrough in the inferolateral right atrium. No other similar case has been reported except a single case report from a Japanese institution [59], probably because three-dimensional electroanatomical activation mapping to identify the site of earliest atrial activation during fast-slow AVNRT has been used by only a few electrophysiologists [60]. Recent anatomical studies have described a right inferior extension of the AV node into the cavo-tricuspid isthmus [56]. The genesis of the inferolateral right atrial SP will be discussed later.

#### 2.2.2 Electrophysiological Diagnosis of Associated Atypical AVNRT

**Fig. 5.**
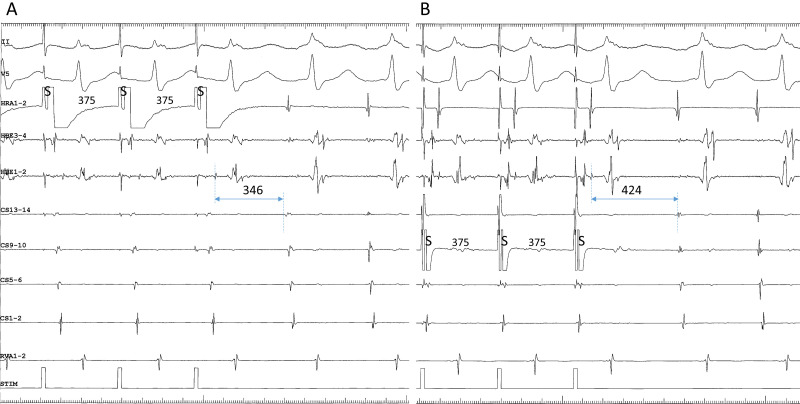
**Differential atrial entrainment pacing at an S-S cycle length of 365 ms from the high right atrium (HRA1-2) (A) and the proximal coronary sinus (CS9-10) (B), in a patient presenting with fast-slow AVNRT using an inferolateral, right atrial SP**. This patient is the same as in Fig. 4D. Note: (1) the atypical atrial activation sequence during tachycardia, characterized by nearly simultaneous atrial electrograms in the HRA and in the His bundle region (HBE1-2); and (2) shorter His-atrial intervals immediately after entrainment pacing (indicated by horizontal bidirectional arrows and numbers above) in the HRA than in the proximal coronary sinus (CS9-10). II, surface electrocardiogram lead II; CS13-14 to 1-2, proximal to distal coronary sinus; RVA, right ventricular apex.

During tachycardia, slightly wider and sometimes biphasic (+/-) P waves in the inferior leads (Figs. 4D,5) [58] is a unique electrocardiographic/intracardiac characteristic of fast-slow AVNRT using an inferolateral right atrial SP as the retrograde limb (inferolateral fast-slow AVNRT), compared to typical fast-slow AVNRT. The atrial activation sequence during inferolateral, fast-slow AVNRT is unique: the site of earliest atrial activation is consistently recorded in the proximal CS; however, the atrial electrogram in the HRA relative to the His bundle region is recorded earlier than during typical, fast-slow AVNRT, due to a lateral atrial breakthrough away from the posterior septum (Figs. 4D,5).

In our experience, inferolateral fast-slow AVNRT is easily diagnosed, using standard criteria [58]. First, a V-A-V response upon ventricular induction/entrainment is often observed [31], probably because retrograde conduction in the lower common pathway is preserved, excluding the diagnosis of atrial tachycardia. Second, a diagnosis of AV reentrant tachycardia using a slowly conducting, posterolateral or posterior accessory pathway as the retrograde limb can be easily excluded by the absence of change in the atrial cycles during the transition zone of the QRS complex, immediately after ventricular entrainment [25, 26]. As mentioned earlier, in a diagnosis of superior type fast-slow AVNRT, we favor ventricular overdrive pacing during tachycardia instead of single ventricular extrastimuli to exclude AV reentrant tachycardia and orthodromic reentrant tachycardia using concealed nodo-ventricular or nodo-fascicular fibers. Single extrastimuli or overdrive pacing from the right ventricular base contralateral to the site of earliest atrial activation may also help in the exclusion of these accessory pathway-mediated tachycardias [29, 61]. Differential ventricular entrainment pacing from the right ventricular apex and base may also be useful to exclude AV reentrant tachycardia [62].

We present a unique response to differential atrial entrainment pacing of inferolateral fast-slow AVNRT. Differential atrial entrainment pacing of typical fast-slow AVNRT may reveal the absence of VA linking, characterized by a shorter VA interval after pacing from the proximal CS than from the HRA, due to the pacing site-dependent effect described earlier [35]. In contrast, differential atrial entrainment pacing of inferolateral, fast-slow AVNRT may reproducibly reveal a reverse relationship of the VA interval, characterized by a shorter VA interval after pacing from the HRA than from the proximal CS (Fig. 5). This may be due to the atypical location of the atrial end of the SP, relatively away from the proximal CS and closer to the HRA, causing a deeper penetration into the SP during pacing from the HRA than from the proximal CS. The subsequent retrograde conduction time over the SP is, therefore, shorter after pacing from the HRA than from the proximal CS. This information may be helpful when interpreting the results from differential atrial entrainment pacing of fast-slow AVNRT.

#### 2.2.3 Catheter Ablation

An inferolateral right atrial SP can be successfully ablated, using the standard techniques applied for typical SP, or at the site of earliest atrial activation [58]. An accelerated junctional rhythm developing during ablation may be due to heating of the AV nodal transitional cells constituting these SP [58, 63]. However, in some patients, the tachycardia may be refractory, requiring the delivery of multiple radiofrequency applications due to shifts of the site of earliest atrial activation during tachycardia after each application [58, 59], similar to the observations made during ablation of superoanterior SP described earlier. This may also indicate the presence of a relatively wide SP with multiple connections into the atrial muscle [58]. Based on these observations, we recommend using the standard technique targeting the posteroseptum to eliminate a right, inferolateral atrial SP as first choice, irrespective of the site of earliest atrial activation, or as alternate choice when ablation at the site of earliest atrial activation is unsuccessful.

In most patients with inferolateral fast-slow AVNRT, low-frequency potentials preceding local atrial electrograms are detectable near the site of earliest atrial activation during tachycardia (Fig. 4D) [58]. These potentials may reflect retrograde activation over an inferolateral right atrial SP [58].

## 3. Classification and Prevalence of Atypical AVNRT Subtypes

AVNRT is usually subclassified in slow-fast, fast-slow or slow-slow subtypes, according to the atrio-His (AH) and His-atrial (HA) interval or the AH/HA ratio, and according to the site of earliest atrial activation in the Koch’s triangle during tachycardia [64]. This is based on the understanding that (a) the atrial ends of the fast and slow pathways are located in the anterior and posterior septum, respectively, within Koch’s triangle, (b) the AH and HA intervals approximate the conduction times over the FP and SP, and (c) the conduction time is longer over the SP than over the FP. However, in some cases, the subtype classification is inconsistent with an actual circuit of AV nodal reentry. Therefore, other investigators have divided AVNRT into typical and atypical subtypes without further specification of the pathways [65].

As discussed earlier, atypical AVNRT using a variant of SP extending into the tricuspid annulus is a diagnosis by exclusion according to standard criteria, and a subsequent identification of the pathways used in the reentry circuit based on the earliest site of atrial activation during tachycardia and the P-QRS relationship or AH/HA ratio (Table 1). An earliest site of atrial activation observed outside Koch’s triangle is firm evidence of the presence of a retrograde SP variant distinct from previous classifications. A retrograde AV nodal pathway should not be identified from the length of the HA interval during tachycardia, since some atypical AVNRT using a superior SP as the retrograde limb may be associated with a short RP due to enhanced conductivity [44]. In contrast, the anterograde limb during tachycardia is generally defined from the P-QRS relationship (or AH/HA ratio): it is a FP in long RP (or AH/HA ratio <1) and a SP in short RP (or AH/HA ratio >1) tachycardia. However, a rare superior-type of fast-slow AVNRT may be associated with a short RP due to slow conduction over the FP [12].

**Table 1.** **Electrocardiographic and electrophysiologic characteristics of atypical AVNRT using variants of slow pathway extending along the tricuspid annulus**.

Subtype	Earliest site of atrial activation	P-QRS relationship (AH/HA ratio)
Atypical AVNRT using a superior or superoanterior slow pathway		
Fast-slow	Non coronary sinus of Valsalva	Long RP (<1), rarely short RP (>1)
Slow-slow	Perihisian region	Short RP (>1) is common
	Superior or superoanterior right atrium along the tricuspid annulus	
Atypical AVNRT using an inferolateral slow pathway		
Fast-slow	Inferior or inferolateral right atrium along the tricuspid annulus	Long RP (<1)

The exact prevalence of atypical AVNRT using variants of SP extending into the tricuspid annulus remains unclear, since no systematic study has been performed prospectively. In our experience, the prevalence of fast-slow AVNRT using a superior SP is not low, and can be expected in >50% of patients with ATP-sensitive long RP tachycardia with an earliest site of atrial activation near the AV node. Fast-slow AVNRT using a superoanterior or inferolateral SP is extremely rare. A <1% prevalence of slow-slow AVNRT using a superior SP as retrograde limb has been reported [45]. It may, however, be higher since this tachycardia may be incorrectly diagnosed as typical slow-fast AVNRT.

## 4. AV Ring Tachycardia Hypothesis

### 4.1 Electrophysiological Similarities between Variants of SP Extending into the Tricuspid Annulus and the Origin of Verapamil- (or Adenosine-) Sensitive Atrial Tachycardias

**Fig. 6.**
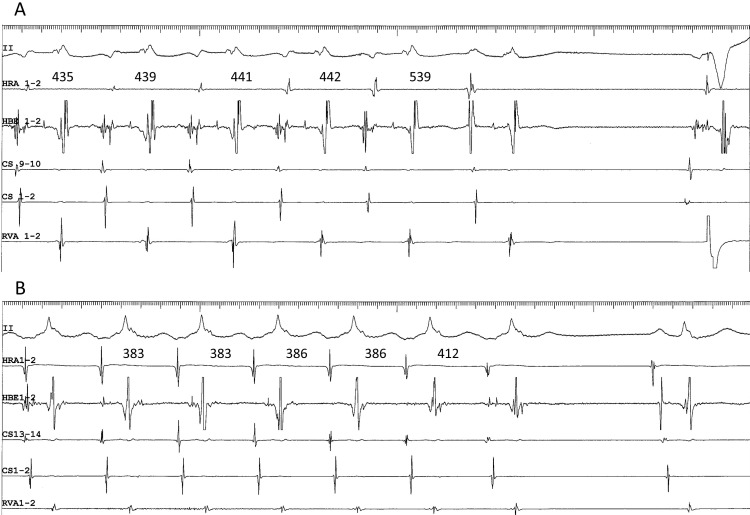
**Termination of superior-type (A) and inferolateral fast-slow (B) AVNRT, following 4- and 10-mg boluses of ATP, respectively**. (A) Tachycardia with the earliest site of atrial activation in the His bundle electrogram (HBE1-2), ending with an ectopic atrial event that followed the interatrial interval of 539 ms, longer than the tachycardia cycle length, suggesting that the tachycardia was terminated by retrograde block (or slowed by a retrograde delay) in the superior SP, immediately before the ectopic atrial cycle. (B) Tachycardia with the earliest site of atrial activation in the proximal coronary sinus (CS13-14), slowing in association with a prolongation of the VA interval, and terminated in an “A no V” ending, suggesting that the termination was due to retrograde block in the right atrial, inferolateral SP. Numbers above the high right atrial (HRA1-2) channel indicate the interatrial intervals. II, surface electrocardiogram lead II; CS13-14 to 1-2, proximal to distal coronary sinus; RVA, right ventricular apex.

As described earlier, we have identified variants of SP extending into the tricuspid annulus, including superior, superoanterior and inferolateral right atrial SP, suggesting that the cells constituting the variants of SP are distributed in the right atrial free wall, all around the tricuspid annulus. It is noteworthy that this distribution of SP tissue resembles that of the origin of verapamil- (or adenosine-) sensitive atrial tachycardia. Adenosine-sensitive atrial tachycardia originating from the vicinity of the AV node is a distinctive form of reentrant atrial tachycardia which can be ablated on the right side of the perihisian region [66, 67, 68, 69], or from the non-coronary sinus of Valsalva [47, 68, 70, 71, 72, 73, 74, 75, 76, 77, 78, 79]. These sites are also preferred to ablate superior SP [12, 33]. The right atrial free wall, along the tricuspid annulus, is also a typical site of verapamil- (or adenosine-) sensitive atrial tachycardia [80, 81, 82, 83]. Its site of origin seems to nearly overlap the putative trajectory of superoanterior or inferolateral right atrial SP. In addition, the characteristics of the local intracardiac electrograms at the site of successful ablation, such as the simultaneous recording of near-field atrial and far-field ventricular activation, and a >1 A:V ratio with any of the tachycardias (Fig. 4), suggests that the arrhythmogenic tissue is distributed inside the atrial muscle adjacent to the tricuspid annulus. Furthermore, these atrial tachycardias and atypical AVNRT seem to share several characteristics. *The first* is adenosine or adenosine triphosphate (ATP) sensitivity of the arrhythmogenic substrate, although the terminating dose of ATP seems to vary among tachycardias. Adenosine-sensitive atrial tachycardia originating from the vicinity of the AV node and from the tricuspid annulus requires 4.0 [66] and 5.0 [83] mg, respectively, to be terminated. In our experience, a superior-type, fast-slow AVNRT requires a mean of approximately 3.0 mg for its termination by retrograde block in the superior SP (Fig. 6A) [12]. The sensitivity of these tachycardias to small doses of ATP is in contrast with that of typical slow-fast AVNRT [84], and may be a characteristic of the arrhythmogenic substrate located in the superior or superoanterior right atrial free wall, along the tricuspid annulus, irrespective of the mechanism of tachycardia. In contrast, a higher average dose of 10.0 mg was required to terminate inferolateral, fast-slow AVNRT (Fig. 6B) [58], like typical slow-fast AVNRT [84].

**Fig. 7.**
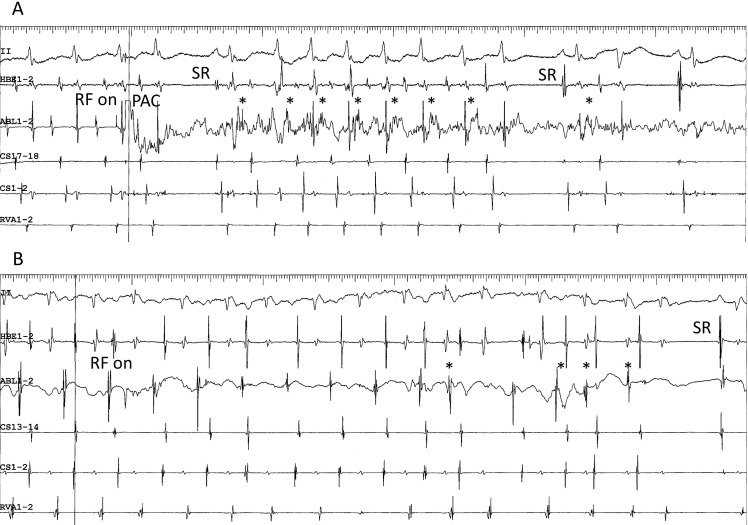
**Development of accelerated ectopic atrial cycles during radiofrequency delivery**. (A) Accelerated ectopic atrial rhythm (asterisks) developing immediately after radiofrequency energy was delivered (RF on) during an ongoing superoanterior-type of fast-slow AVNRT, in the same patient as shown in Fig. 4C. The tachycardia is terminated by a premature atrial complex (PAC) immediately after the delivery of radiofrequency energy. (B) ATP-sensitive atrial tachycardia originating from the inferolateral right atrium along the tricuspid annulus, followed by return to sinus rhythm (SR) in the same patient as in Fig. 4E. II, surface electrocardiogram lead II; HRA1-2, high right atrium; HBE1-2, His bundle electrogram; CS13-14 to 1-2, proximal to distal coronary sinus; RVA1-2, right ventricular apex.

*The second* characteristic is the detection of low-frequency potentials preceding the local atrial activation, near the site of successful ablation. As described earlier, these potentials are found in the superoanterior or inferolateral fast-slow AVNRT (Fig. 4C,D, respectively) and may reflect retrograde activation across the SP [58]. Although the presence of prepotentials during adenosine-sensitive atrial tachycardia has not been reported previously [67, 80, 85], we have reproducibly detected low-frequency prepotentials within a localized area near the site of earliest atrial activation during an atrial tachycardia originating from the inferolateral right atrium, along the tricuspid annulus (Fig. 4E). Yamabe *et al*. [86] have described low-frequency, late potentials *following* local atrial activation during sinus rhythm, probably reflecting a delayed activation of the tachycardia origin, near the site of successful ablation, in patients presenting with atrial tachycardia originating from the tricuspid annulus. The presence of these potentials suggests a slowly conducting, common arrhythmogenic substrate of these tachycardias. *The third* characteristic is the development of accelerated atrial ectopic activity during delivery of radiofrequency energy. This is often observed during ablation of right atrial, superior, superoanterior (Fig. 7A) and inferolateral SP, as described earlier. Moreover, in contrast to previous reports [80, 86], we have also observed it during ablation of atrial tachycardia originating from the tricuspid annulus (Fig. 7B). This activity may be caused by heating of the AV nodal transitional cells present in these tissues [63]. *The fourth *characteristic is the occurrence of multiple shifts of the site of earliest atrial activation during tachycardia with each radiofrequency application. As described earlier, this phenomenon is occasionally observed after the ablation of superoanterior or inferolateral right atrial SP, or of atrial tachycardia originating from the tricuspid annulus [69]. Collectively, these anatomical, electrophysiological and pharmacological characteristics suggest that the AV node-like tissue distributed around the tricuspid annulus is the arrhythmogenic substrate shared by these tachycardias.

**Fig. 8.**
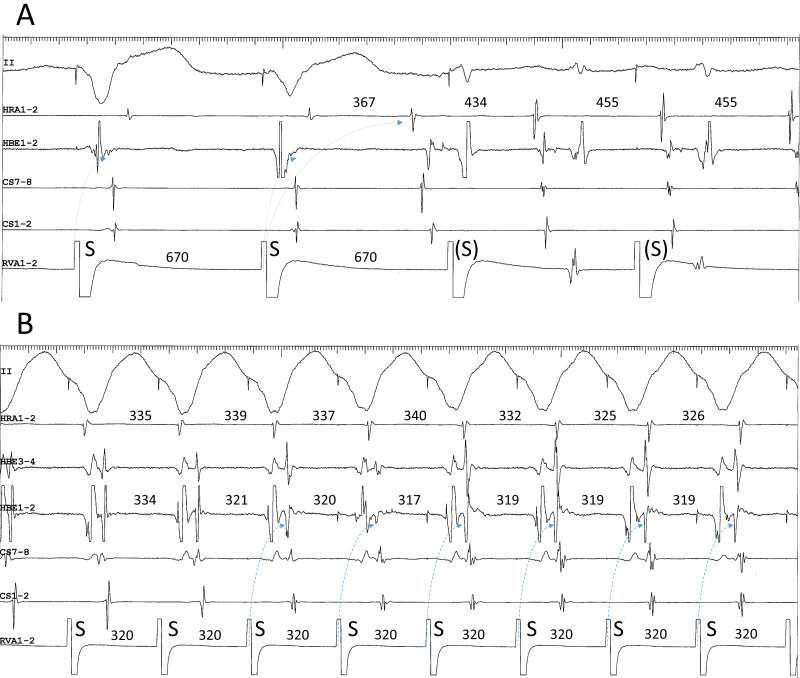
**Example of superoanterior type fast-slow AVNRT (A) followed by the development of atrial tachycardia (B) originating from the superoanterior right atrium, along the tricuspid annulus, after ablation of a superoanterior SP**. This is the same patient as in Fig. 4C. (A) Induction of superior-type of fast-slow AVNRT by ventricular overdrive pacing at an S-S cycle length of 670 ms. Retrograde conduction via a FP is present after the 1st and 2nd stimuli, with an earliest site of atrial activation in the His bundle region (HBE1-2). Immediately after the 2nd stimulus, a long RP tachycardia with a site of earliest atrial activation in the high right atrium (HRA1-2) is induced, after a V-A-A-V response where the 367-ms interatrial interval is 88 ms shorter than the 455-ms tachycardia cycle length, consistent with a dual atrial response from simultaneous retrograde conduction (dotted arrows) over the FP and the superoanterior SP. The 3rd and 4th stimuli (S), do not capture the ventricles. (B) Ventricular entrainment pacing at an S-S cycle length of 320 ms during ongoing atrial tachycardia with a site of earliest atrial activation in the HRA. Ventricular overdrive pacing captures the atrial electrogram via the FP in HBE1-2 (dotted arrows) after the 3rd stimulus, without capture of the atrial electrogram in HRA1-2, suggesting intraatrial fusion of a retrograde wavefront originating from the FP, with an atrial wavefront propagating from the site of origin of the atrial tachycardia. II, surface electrocardiogram lead II; HRA1-2, high right atrium; HBE1-2, His bundle electrogram; CS7-8 to 1-2, proximal to distal coronary sinus; RVA1-2, right ventricular apex.

We observed the noteworthy case of a 68-year-old man who developed ATP-sensitive atrial tachycardia late after ablation of a superoanterior SP mediating a superoanterior-type of fast-slow AVNRT (Figs. 4C,8). The 12-lead electrocardiogram during tachycardia revealed the presence of a long RP interval, with a biphasic P wave in the inferior leads (Fig. 4C). The tachycardia was induced with ventricular stimulation following a dual atrial response (Fig. 8A) [30]. Fast-slow AVNRT was diagnosed after the exclusion of AV reentry by the observation of VA dissociation during ventricular overdrive pacing of the tachycardia, which required 12 mg of ATP for its termination. Electroanatomical activation mapping of the right atrium during ongoing tachycardia revealed a site of earliest atrial activation in the superoanterior right atrium along the tricuspid annulus (Fig. 4C). The successful ablation of the tachycardia by delivery of radiofrequency energy at or near that site (Fig. 4C) was followed by a brief run of accelerated ectopic atrial rhythm (Fig. 7A). The long RP tachycardia recurred six months later, with a P wave morphology and polarity, and site of earliest atrial activation like those observed before the ablation procedure. However, in contrast to the fast-slow AVNRT present before ablation, ventricular entrainment pacing of the tachycardia revealed the presence of intraatrial fusion (Fig. 8B), confirming the diagnosis of atrial tachycardia [87]. Moreover, the dose of ATP required to terminate the recurrent tachycardia had decreased to 2.0 mg, suggesting a change in the electrophysiological properties of the substrate. A successful second ablation procedure was performed at or near the site of earliest atrial activation. In this case, we hypothesize that the common, ATP-sensitive, arrhythmogenic tissue was the superoanterior SP and origin of the atrial tachycardia, and that the latter might be an atrial remnant of SP isolated by the ablation.

### 4.2 The Arrhythmogenic AV Ring Substrate

The presence of calcium channel-dependent atrial tissue along the tricuspid annulus was already suspected to be the arrhythmogenic substrate of verapamil- or adenosine-sensitive atrial tachycardias based on the pharmacological responses described in the original report [83]. This hypothesis is supported by basic research. In histological studies, Anderson *et al*. [88, 89, 90] found specialized atrial tissue sporadically distributed around adult, human tricuspid valves, thought to be embryonic remnants of AV ring tissue. McGuire *et al*. [91, 92] described a superficial ring of cells around the tricuspid annulus histologically similar to atrial myocytes. However, their “nodal-like” electrophysiologic characteristics, response to adenosine, and absence of connexin43 suggest that these cells may be an arrhythmogenic substrate [91, 92]. Importantly, AV ring tissue encircling the tricuspid and mitral annulus, including in adult hearts, has been observed in several immunohistochemical studies [93, 94, 95, 96]. Other basic experiments in embryonic mouse hearts have observed the development of AV ring reentry associated with spatial dissociation of anterograde and retrograde conduction [97]. Therefore, the AV ring tissue may be viewed as a potential arrhythmogenic substrate of atrial tachyarrhythmias originating around the tricuspid annulus. On the other hand, the retroaortic node may be the origin of adenosine-sensitive atrial tachycardia originating from near the AV node [98]. This well-known, abundant, nodal-like tissue located near the rings encircling the tricuspid and mitral annuli (Fig. 1B), is found in the interatrial septum, behind to non-coronary sinus of Valsalva, precisely where adenosine-sensitive atrial tachycardias have been ablated in humans [98].

During embryological development of the heart, the AV node originates from the AV ring [95, 99, 100]. Based on histological observations in fetal and adult hearts [88, 89, 90], a larger segment of AV ring was considered to disappear during the process, with only the compact node and inferior extensions remaining mostly localized within Koch’s triangle [8, 101]. However, several immunohistochemical studies cited earlier revealed that the rings surrounding the tricuspid and mitral annuli are continuous with inferior nodal extensions, including in adult hearts, forming figure-of-eight-shaped rings of nodal and transitional cells [93, 94, 95, 96]. This continuity between the AV node and the AV ring tissues may be explained by the same embryological origin of the AV ring and AV node, i.e., the embryonic AV canal [95, 99, 100]. Based on these basic experiments and our clinical observations, we hypothesize that the AV ring tissue connected to the right inferior extension participates in the conduction across inferolateral SP (Fig. 1B). Furthermore, the origins of the superior or superoanterior SP can also be explained by the AV ring hypothesis. In the original descriptions, the compact AV node had no superior connections with the AV ring tissue, because of the fibrotic tissue present between the retroaortic and the compact nodes [93]. However, a recent study confirmed the existence of an anatomical connection between these nodes [102], suggesting a possible anatomical continuity between compact node and retroaortic node or superior AV ring tissue, operating as the superior or superoanterior SP (Fig. 1B).

Accordingly, we hypothesize that (1) the primitive form of SP variants extending to the tricuspid annulus is created mainly by the retroaortic node or the AV ring tissue, or both (Fig. 1B), and (2) these tissues are shared to form the common arrhythmogenic substrate of superior, superoanterior or inferolateral fast-slow AVNRT and atrial tachycardias originating from near the AV node or along the tricuspid annulus. In addition, acquired factors, including advanced age and hemodynamic overload of the right atrium may promote a structural or electrophysiological remodeling, including fibrotic longitudinal dissociation of the AV ring tissue [97], creating the properties of the SP variant or the origin of atrial tachycardias.

### 4.3 Significance of a Proposed AV Ring Tachycardia

In state-of-the art clinical electrophysiology, AVNRT and atrial tachycardia are classified separately, and are diagnosed and treated according to their electrophysiological mechanisms. However, the etiological relationship between these two tachycardias has not been evoked. We are introducing “AV ring tachycardia” a new, inclusive appellation of atypical AVNRT (Fig. 1B) and atrial tachycardia sharing the AV ring tissue as their common arrhythmogenic substrate. This is our first proposal to adopt these tachycardias and their shared arrhythmogenic substrate, which represents a pharmacological and non-pharmacological interventional target. The promotion of this concept has the following putative clinical implications:

*First*, a deeper understanding of the pathophysiology of the tachycardias from the new perspective of the AV ring tissue as a common arrhythmogenic substrate, including factors determining the development of each tachycardia, and the similarities or differences in their natural history, anatomical structures of the substrate, and electrophysiological and pharmacologic properties. *Second*, a new diagnostic strategy aimed at suppressing the mechanism of AV ring tachycardia. Surprisingly, no uniform criteria are currently used in the differential diagnosis of AV ring tachycardias. The revisiting of these criteria may highlight their challenges and limitations and, perhaps, clarify the characteristics of a tachycardia that could be classified neither as AVNRT nor as atrial tachycardia and, consequently, remained diagnostically obscure. These, in turn, could help in the creation of a new diagnostic scheme or of uniform diagnostic criteria toward the differential diagnosis of AV ring tachycardias. *Third* is an accumulation of knowledge regarding the AV ring which has never been studied clinically. In our experience, high-resolution electroanatomical mapping enables the visualization of the AV ring tissue activation, which might clarify its location and lead to the discovery of new strategies for the ablation of the AV ring tachycardias “substrate” [103].

## 5. Limitations of Our Study

*First*, a few patients with AV reentrant tachycardia using a slowly conducting, concealed accessory pathway may have been included in this study population due to an incompletely exclusive diagnosis. However, few such accessory pathway localized in the anterior septum has been previously reported [104, 105, 106, 107, 108, 109]. *Second*, since we did not directly compare the electrophysiological characteristics of AVNRT using variants of SP with those of atrial tachycardia originating from the tricuspid annulus, the similarities versus dissimilarities of these tachycardias remain uncertain. *Third*, the locational relationship between superior SP and surrounding structures including retroaortic node, non-coronary aortic sinus of Valsalva and tricuspid annulus need to be more precisely localized.

## 6. Conclusions

This review summarized the electrophysiological characteristics and catheter ablation of SP variants found along the tricuspid annulus and associated AVNRT. Based on the similar anatomical, electrophysiological and electro-pharmacological characteristics of these SP and those of atrial tachycardia originating from the tricuspid annulus, as well as on recent basic research reporting the presence of AV node-like ring tissue encircling the annuli, we propose the term “AV ring tachycardia” to define the tachycardias sharing the AV ring tissue as a common arrhythmogenic substrate. This review will help the readers recognize rare types of SP variants and associated AVNRT, and diagnose and cure these complex tachycardias. We hope, with this proposal of a unified tachycardia designation, to open a new chapter in clinical electrophysiology.

## Abbreviations

ATP, adenosine triphosphate; AV, atrioventricular; CS, coronary sinus; FP, fast pathway; HRA, high right atrium; NRT, nodal reentrant tachycardia; SP, slow pathway; VA, ventriculoatrial.
